# Antifungal spectrum of cyclobutrifluram and multi-point mutations in CcSdh proteins confer resistance in *Corynespora cassiicola*

**DOI:** 10.1007/s44154-025-00251-8

**Published:** 2025-09-01

**Authors:** Xinchang Hao, Yiwen Li, Zhaoyue Hang, Yue Chen, Yidong Tang, Jianqiang Miao, Qin Peng, Xili Liu

**Affiliations:** 1 State Key Laboratory for Crop Stress Resistance and High-Efficiency Production, College of Plant Protection, Northwest A&F University, Yangling, 712100 Shaanxi China; 2 Key Laboratory of Plant Protection Resources and Pest Management of Ministry of Education, Key Laboratory of Integrated Pest Management On Crops in Northwestern Loess Plateau of Ministry of Agriculture and Rural Affairs, College of Plant Protection, Northwest A&F University, Yangling, 712100 Shaanxi China; 3 Department of Plant Pathology, College of Plant Protection, China Agricultural University, 2 Yuanmingyuanxi Road, Beijing, 100193 China

**Keywords:** Cyclobutrifluram, *Corynespora cassiicola*, Resistance risk, Point mutation, Resistance mechanism

## Abstract

Cucumber target spot, a major disease that threatens cucumber production, is caused by *Corynespora cassiicola*. Cyclobutrifluram, a novel succinate dehydrogenase inhibitor (SDHI) developed by Syngenta, has demonstrated strong inhibitory activity against various plant pathogenic fungi and nematodes. However, its antifungal spectrum, resistance risk as well as underlying mechanisms of resistance in *C. cassiicola* remain poorly understood. In this study, cyclobutrifluram exhibited potent inhibitory activity against anamorphic fungi and selected ascomycetes, with the mean sensitivity of *C. cassiicola* isolates to the fungicide being 0.98 ± 1.26 μg/mL. Additionally, five laboratory-derived cyclobutrifluram-resistant mutants showed comparable or lower biological fitness than their respective parental isolates. The resistant mutants and field isolates were also found to possess nine distinct point mutations in the *CcSdhB*, *CcSdhC* or *CcSdhD* genes. Finally, cyclobutrifluram exhibited positive cross-resistance with other SDHIs, with the resistance levels varying depending on the specific mutations present. In conclusion, cyclobutrifluram was found to be effective against anamorphic fungi and selected ascomycetes. *C. cassiicola*’s risk of resistance development to cyclobutrifluram was assessed as moderate to high and was primarily associated with mutations in *CcSdh* genes.

**Supplementary Information:**

The online version contains supplementary material available at 10.1007/s44154-025-00251-8.

## Highlights

The antifungal spectrum of cyclobutrifluram was investigated.

*C. cassiicola*’s risk of resistance development to cyclobutrifluram was classified as moderate to high.

Specific point mutations in *CcSdh* genes conferred resistance to cyclobutrifluram.

**Supplementary Information:**

The online version contains supplementary material available at 10.1007/s44154-025-00251-8.

## Introduction

Cucumber is a highly important vegetable crop in China (Liu et al. 2017), and in 2022, its cultivation area covered approximately 1.31 million hectares, yielding a total production of 77.3 million tons (https://www.fao.org/faostat/en/#home). Although cucumbers are affected by numerous diseases, the prevalence and impact of specific pathogens vary according to the cultivation area and cropping history (Zhao et al. 2021). Among these, *Corynespora cassiicola* (Berk. & M. A. Curtis) C.T. Wei, the causal pathogen for cucumber target spot, has become particularly significant (Fischer et al. 2022; Li et al. 2019). *C. cassiicola* is a destructive fungal pathogen with a broad host range (Li et al. 2020), affecting over 530 plant species across 380 genera (Dixon et al. 2009). Notably, its hosts include economically important crops, such as soybean, cotton, tomato, eggplant, cucumber and rubber, causing severe economic losses in over 70 countries (Aumentado and Balendres 2022; Moo-Koh et al. 2022; Oktavia et al. 2021; Rahman et al. 2010; Rondon and Lawrence 2021). In cucumbers, the pathogen primarily infects leaves during the seedling stage and can also attack fruits and stems in mature plants, leading to target spot symptoms characterized by leaf necrosis, defoliation and fruit rot (Li et al. 2012). From 2005 to 2019, the incidence and severity of cucumber target spot have significantly increased in major cucumber-producing regions of China, including Hunan, Hebei and Shandong provinces (Liu et al. 2019; Zhu et al. 2019). Consequently, this disease has become a major constraint for cucumber cultivation, particularly in commercial greenhouse production systems (Duan et al. 2019).

Due to the absence of resistant cucumber cultivars, chemical fungicides remain the primary means of managing cucumber target spot. In China, several classes of fungicides, such as sterol demethylation inhibitors (DMIs), succinate dehydrogenase inhibitors (SDHIs), quinone outside inhibitors (QoIs) and protective fungicides, are approved for controlling this disease (http://www.chinapesticide.org.cn). However, the intensive and repeated use of those chemicals has led to widespread resistance in *C. cassiicola* populations (Miyamoto et al. 2009, 2010). For instance, 87% of the 920 *C. cassiicola* isolates obtained from various regions of China were found to be resistant to boscalid (Zhu et al. 2019), with field resistance to pyraclostrobin also reported (Li 2021). Therefore, there is an urgent need to develop and implement new fungicidal agents to effectively manage cucumber target spot and safeguard cucumber production as well as food security.

SDHIs represent a class of fungicides renowned for their high efficacy and low toxicity (Yin et al. 2024). These compounds exert their antifungal activity by binding to the active site of succinate dehydrogenase, a key enzyme within the tricarboxylic acid cycle, thereby disrupting the respiratory electron transport chain and impairing cellular respiration (Ackrell 2000; Huang and Millar 2013). Since the introduction of the first SDHI fungicide, carboxin, 24 SDHIs have been commercialized to date, as reported by the Fungicide Resistance Action Committee (FRAC) (https://www.frac.info/home). However, despite the effectiveness, the widespread and frequent application of SDHIs, coupled with their structural similarity, has led to growing concerns about the development of resistance (Cheng et al. 2024). In this context, the primary mechanism leading to SDHI resistance often involves point mutations in the SdhB, SdhC and SdhD subunits of succinate dehydrogenase, which result in reduced or abolished binding affinity for the fungicides (Horsefield et al. 2006).

Cyclobutrifluram, developed by Syngenta, was initially introduced as a nematicide (Tsukamoto et al. 2021), but in recent years, several patents filed in China have described the use of cyclobutrifluram in combination with other fungicides for nematode control (Brown and Faske 2024; Liu et al. 2024). Additionally, cyclobutrifluram has also demonstrated potent antifungal activity against several *Fusarium* species (Li et al. 2024), such as *F. graminearum* (Miao et al. 2024), *F. fujikuroi* (Xue et al. 2023) and *F. pseudograminearum* (Li et al. 2023). While FRAC classifies SDHIs as having a medium to high risk for resistance development, the antifungal spectrum of cyclobutrifluram and the potential for resistance development in *C. cassiicola* are yet to be reported. Hence, the current research sought to: (i) assess the antifungal spectrum of cyclobutrifluram; (ii) investigate the sensitivity of *C. cassiicola* to cyclobutrifluram; (iii) determine the risk of resistance development in *C. cassiicola*; and (iv) elucidate the mechanisms underlying *C. cassiicola*’s resistance to cyclobutrifluram.

## Results

### Cyclobutrifluram’s antifungal spectrum

**Fig. 1:**
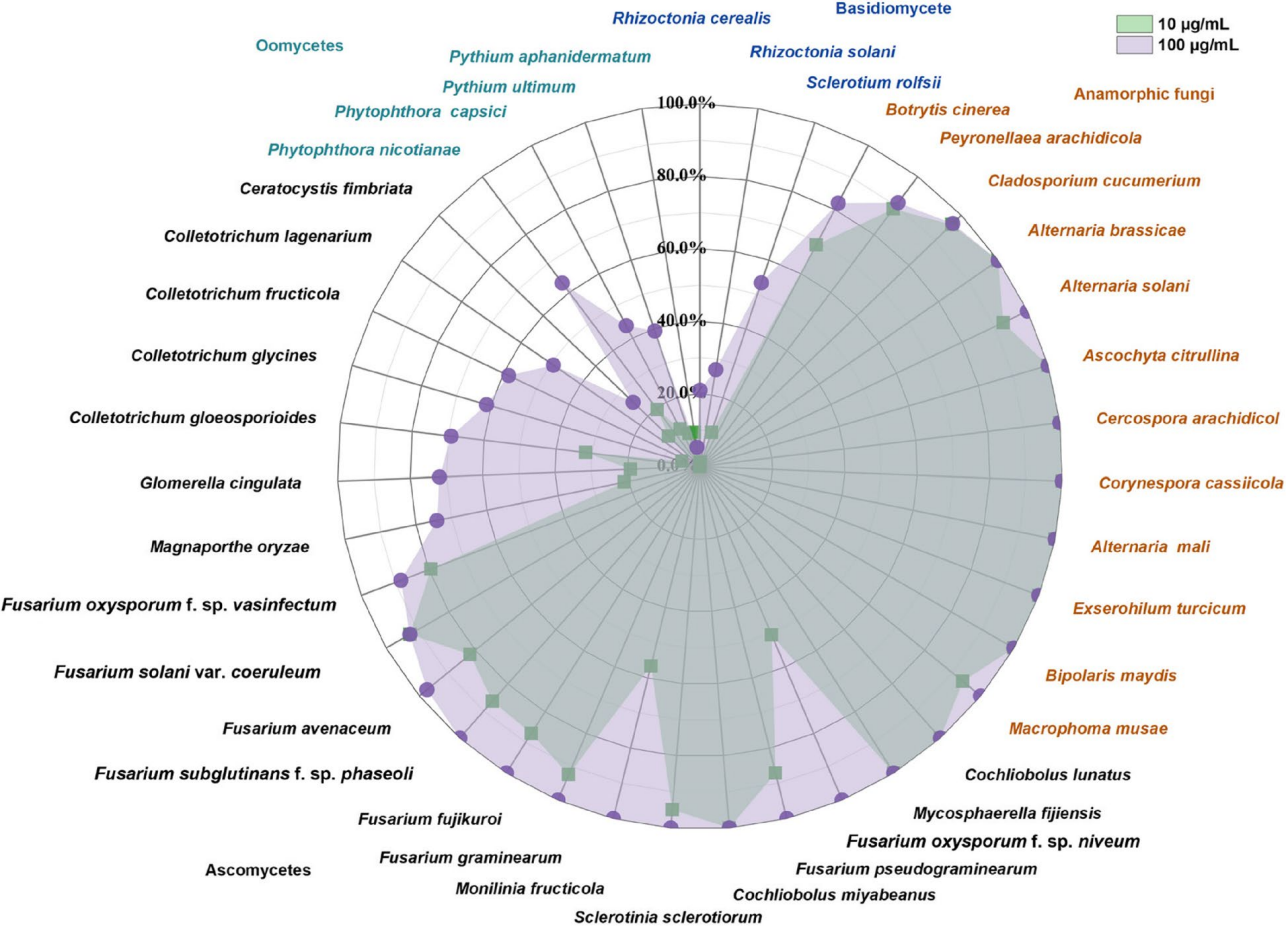
In vitro inhibitory activity of cyclobutrifluram against plant pathogens

Cyclobutrifluram displayed strong inhibitory activity against a broad range of anamorphic fungi, including *Bipolaris maydis*, *Macrophoma musae*, *Exserohilum turcicum*, *Cladosporium cucumerinum*, *Alternaria brassicae*, *Alternaria solani*, *Corynespora cassiicola*, *Ascochyta citrullina* and *Botrytis cinerea*. Additionally, certain Ascomycota species, such as *Fusarium* spp., *Cochliobolus miyabeanus*, *Cochliobolus lunatus* and *Mycosphaerella fijiensis*, were markedly inhibited by 10 and 100 µg/mL of cyclobutrifluram. Overall, the EC_50_ values for these pathogens varied between 0.0042 and 0.9663 µg/mL (Table 1). However, cyclobutrifluram exhibited relatively low inhibitory activity against other Ascomycota species not listed above as well as against pathogens from the phyla Basidiomycota and Oomycota (Fig. 1).

**Table 1:** In vitro inhibition of plant pathogens by cyclobutrifluram

Phylum	Isolates	Y = *a* + *b*X^*^	^#^*R*	EC_50_ (μg/mL)
Ascomycota	*Cochliobolus miyabeanus*	Y = 9.2076 + 2.9420X	0.9738	0.0371 (0.0278–0.0495)
	*Mycosphaerella fijiensis*	Y = 5.4743 + 0.8474X	0.9124	0.2756 (0.0645–1.1771)
	*Cochliobolus lunatus*	Y = 6.1758 + 0.6716X	0.9568	0.0178 (0.0113–0.0279)
Anamorphic fungi	*Bipolaris maydis*	Y = 6.1110 + 1.4226X	0.9680	0.1656 (0.0799–0.3429)
	*Macrophoma musae*	Y = 7.2200 + 2.3803X	0.9841	0.1168 (0.0782–0.1743)
	*Exserohilum turcicum*	Y = 6.2226 + 1.9413X	0.9962	0.2346 (0.1736–0.3170)
	*Cladosporium cucumerium*	Y = 8.1133 + 2.8890X	0.9851	0.0836 (0.0609–0.1148)
	*Alternaria brassicae*	Y = 10.8740 + 1.9285X	0.9985	0.0090 (0.007–0.0011)
	*Peyronellaea arachidicola*	Y = 6.8852 + 1.1334X	0.9500	0.0217 (0.0131–0.0360)
	*Botrytis cinerea*	Y = 5.0092 + 0.6174X	0.9379	0.9663 (0.2239–4.1712)
	*Alternaria solani*	Y = 5.2968 + 0.4856X	0.9140	0.2448 (0.0593–1.0117)
	*Corynespora cassiicola*	Y = 6.0729 + 0.6759X	0.9710	0.0259 (0.0174–0.0383)
	*Ascochyta citrullina*	Y = 10.5607 + 2.3411X	0.9594	0.0042 (0.0019–0.0095)

EC_50_, effective concentration for 50% growth inhibition of mycelial growth

^*^ X in the linear regression is the logarithm of fungicide concentration, and Y is the odds value corresponding to the inhibition rate

^#^ *R* is the correlation coefficient

### *C. cassiicola*’s sensitivity to cyclobutrifluram

**Fig. 2:**
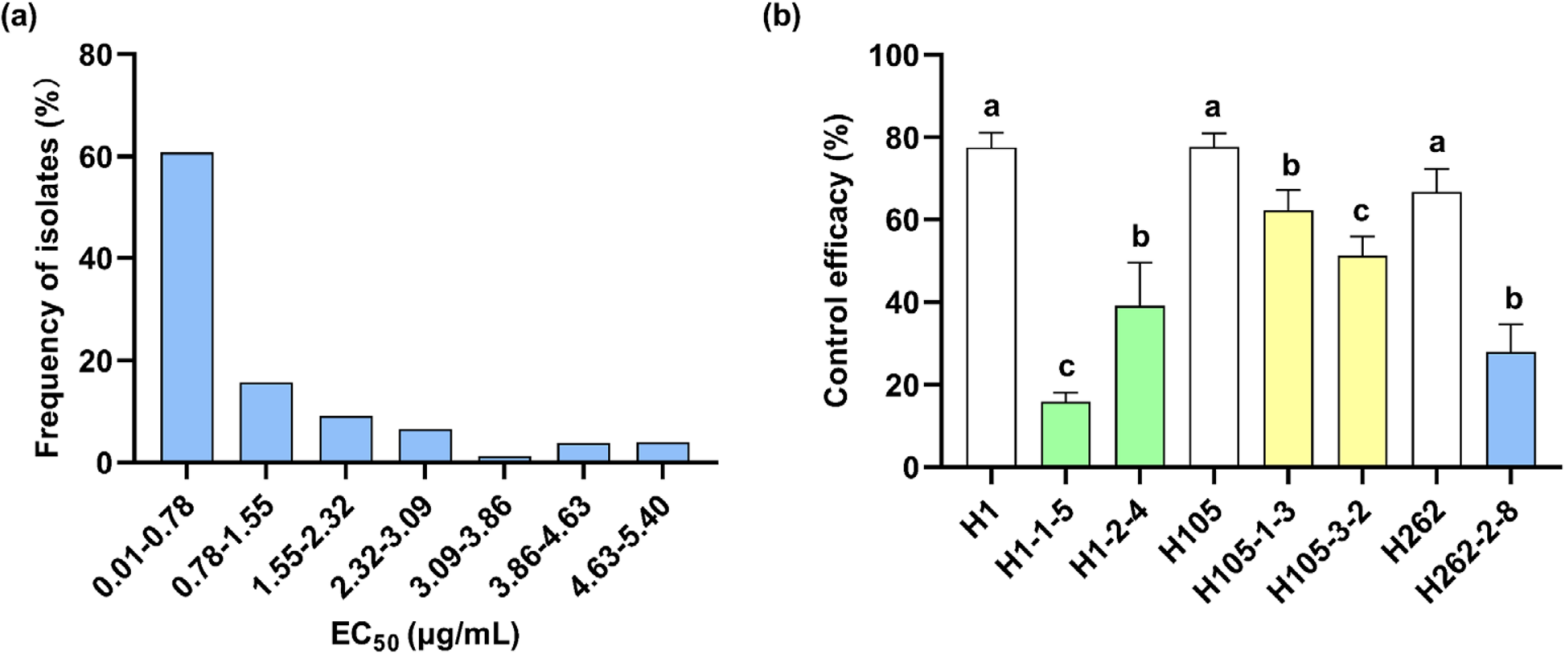
Combined analysis of cyclobutrifluram: sensitivity distribution of *Corynespora cassiicola* isolates and control efficacy against cucumber target spot. **a** Sensitivity distribution of 171 *Corynespora cassiicola* isolates to cyclobutrifluram. **b** Control efficacy of cyclobutrifluram against cucumber target spot caused by *Corynespora cassiicola*. H1-1-5, H1-2-4, H105-1-3, H105-3-2, and 262-2-8 are cyclobutrifluram-resistant mutants, while H1, H105, and H262 are the corresponding parental isolates

In this study, all 171 *C. cassiicola* isolates exhibited significant variations in their sensitivity to cyclobutrifluram, with their EC_50_ values ranging from 0.0071 to 58.70 μg/mL (Fig. S1). This wide range suggested the presence of subpopulations with reduced sensitivity as well as naturally occurring resistant isolates in the field. To refine the dataset, outliers were then excluded using SPSS software, and after filtering, 153 isolates were retained for further analysis, with their mean EC_50_ being 0.98 ± 1.26 μg/mL (Fig. 2a).

### Characterization of *C. cassiicola* mutants exhibiting cyclobutrifluram resistance

#### Resistance level and stability

Five highly resistant mutants (H1-1-5, H1-2-4, H105-1-3, H105-3-2 and H262-2-8) were obtained from three sensitive parental isolates (H1, H105 and H262) through fungicide adaptation, with these mutants exhibiting resistance factors (RFs) exceeding 480 (Table 2). After ten successive subculturing onto fungicide-free PDA, the tenth generation displayed significantly lower resistance levels. Specifically, H1-1-5 and H1-2-4 showed moderate resistance, while H105-1-3, H105-3-2 and H262-2-8 retained high resistance levels (Table 2).

**Table 2:** Resistance level and stability of cyclobutrifluram-resistant mutants of *Corynespora cassiicola*

Isolate	Type	Mutation in CcSdh proteins	EC_50_ (μg/mL)		RF ^a^		FSC ^b^
			First	Tenth	First	Tenth	
H1	Parent	—	0.014	0.022	—	—	—
H1-1-5	Mutant	CcSdhC^H134Q^	6.820	0.870	487.14	39.54	0.08
H1-2-4	Mutant	CcSdhC^H134Q^	7.610	0.910	543.57	41.36	0.08
H105	Parent	—	0.018	0.014	—	—	—
H105-1-3	Mutant	CcSdhB^H278Y^	18.890	4.060	1049.44	290.00	0.28
H105-3-2	Mutant	CcSdhC^S135R^	23.780	3.570	1321.11	255.00	0.19
H262	Parent	—	0.008	0.011	—	—	—
H262-2-8	Mutant	CcSdhB^H278Y^	13.290	7.960	1772.00	723.63	0.41

^a^ RF (resistance factor), the ratio of the EC_50_ of mutants relative to the EC_50_ of their parental isolate

^b^ FSC (factor of sensitivity change), the ratio of the RF of the tenth transfer to that of the first

### Effects of temperature on mycelial growth

The isolates exhibited varying colony diameters across the tested temperatures, and in both the sensitive isolates and resistant mutants, the 25−30℃ range was identified as optimal for mycelial growth (Table S6). In particular, the resistant mutants displayed similar or greater mycelial growth rates than their parental isolates at most temperatures. The only exceptions were mutants H1-1-5 and H105-3-2 for which the growth rates were reduced compared with their corresponding parental isolates (Table S6).

### Conidial production, germination rates and pathogenicity

The resistant mutants showed similar or lower conidial production compared with their corresponding parental isolates, with mutant H262-2-8 being an exception (Table 3). Similarly, germination rates of conidia from resistant mutants were generally comparable to or significantly lower than those of their parental isolates, except for mutant H105-3-2, which showed a higher germination rate than its parent (Table 3). Subsequent assays revealed that the resistant mutants showed markedly reduced pathogenicity compared with the parental isolates, with the exception of mutant H105-1-3 for which enhanced pathogenicity was observed (Table 3). Furthermore, at 50 µg/mL of cyclobutrifluram, significantly higher disease control efficacy was achieved for the parental isolates in comparison with the resistant mutants (Fig. 2b). Finally, the resistant mutants’ overall biological fitness was determined by calculating their compound fitness index (CFI). In this case, all resistant mutants exhibited significantly lower CFIs compared with their parental isolates, with the exception of H105-1-3, which had a higher CFI than its parental isolate (Table 3).

**Table 3:** Fitness of cyclobutrifluram-resistant mutants and their parental isolates of Corynespora cassiicola^*^

Isolate	Type	Colony diameter at 28℃ (cm)	Conidia production (× 10^3^/mL)	Conidia germination (%)	Lesion area (mm^2^)	CFI ^#^ (× 10^9^)
H1	Parent	6.94 b	10.67 a	85.12 a	302.80 a	1.90 a
H1-1-5	Mutant	5.87 c	5.53 b	71.73 b	180.33 c	0.42 c
H1-2-4	Mutant	7.56 a	6.67 b	86.94 a	194.33 b	0.85 b
H105	Parent	7.76 a	16.67 a	87.55 b	366.34 b	4.15 b
H105-1-3	Mutant	7.71 a	18.67 a	89.40 b	429.55 a	5.53 a
H105-3-2	Mutant	5.51 b	5.33 b	94.36 a	111.43 c	0.31 c
H262	Parent	7.78 a	5.00 b	83.44 a	484.20 a	1.57 a
H262-2-8	Mutant	7.83 a	6.00 a	81.92 a	260.57 b	1.00 b

^*^ Means within a column followed by the same letter are not significantly different based on Tukey’s HSD test (*P* < 0.05)

^#^ CFI (compound fitness index) = colony diameter × conidia production × conidia germination × lesion area

### Cross-resistance

**Fig. 3:**
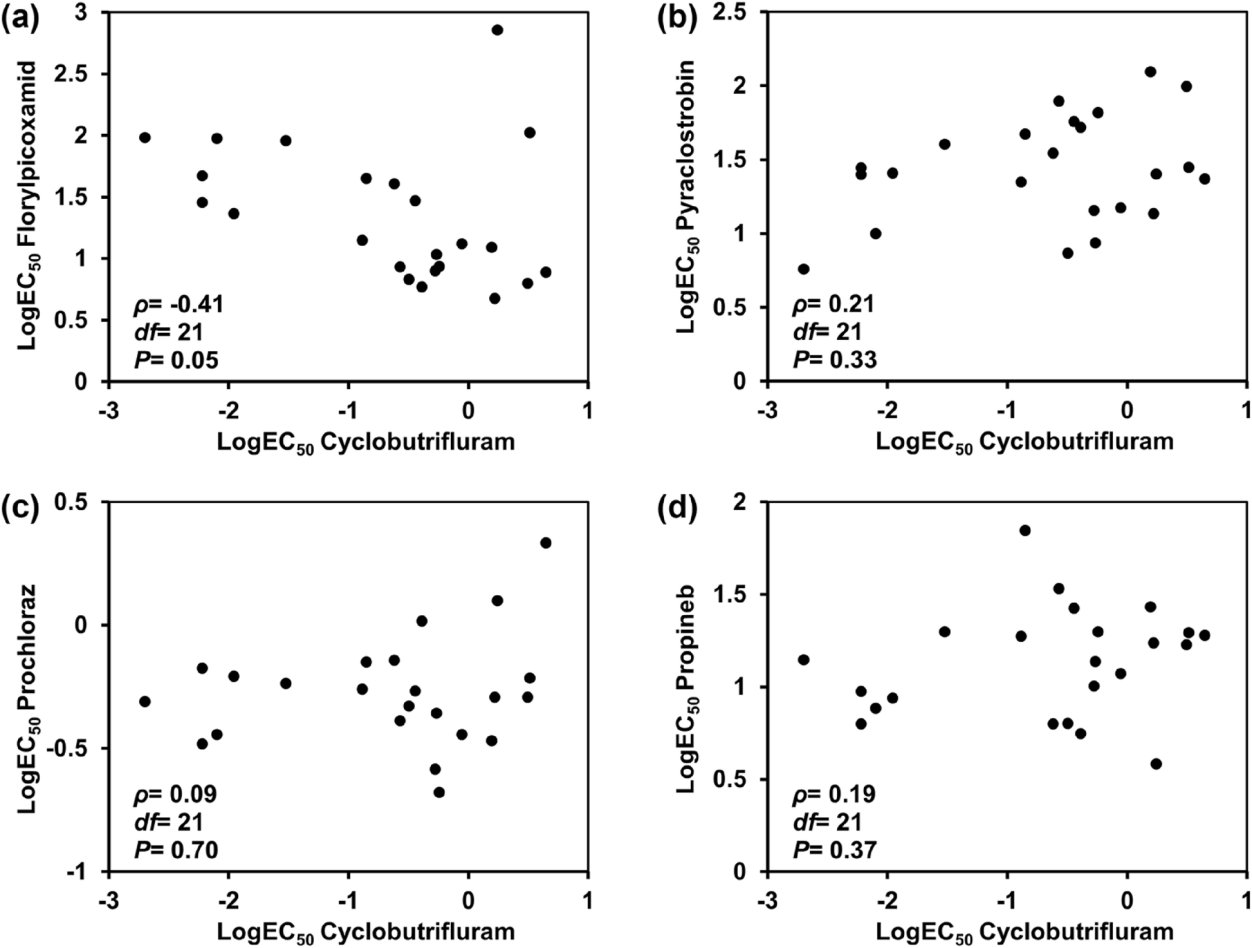
Cross-resistance between cyclobutrifluram and other fungicides used to control cucumber target spot: (**a**) florylpicoxamid, (**b**) pyraclostrobin, (**c**) prochloraz, and (**d**) propineb

Cross-resistance between two fungicides was categorized based on Spearman's rank correlation coefficient (*P* < 0.05) as follows: none (*ρ* < 0.3), low (0.3 ≤ *ρ* < 0.5), medium (0.5 ≤ *ρ* < 0.8) and strong (*ρ* > 0.8). Sensitivity testing and analysis of Spearman’s correlation coefficients, it was found that cyclobutrifluram did not show cross-resistance (*P* > 0.05) with florylpicoxamid, pyraclostrobin, prochloraz or propineb (Fig. 3).

### *CcSdh* genes expression with or without cyclobutrifluram

When cyclobutrifluram was absent, the expression level of *CcSdh* genes in the resistant mutants was not markedly different from that of their corresponding sensitive parental isolates (Fig. S2). Similarly, *CcSdhA*_*1*_, *CcSdhA*_*2*_ and *CcSdhB* expression in the resistant mutants remained comparable to that of the parental isolates upon exposure to cyclobutrifluram. However, the resistant mutants exhibited significantly lower *CcSdhC* and *CcSdhD* expression relative to their sensitive parental isolates (Fig. S2).

### Cloning and analysis of *CcSdh* genes from cyclobutrifluram-resistant *C. cassiicola*

**Fig. 4:**
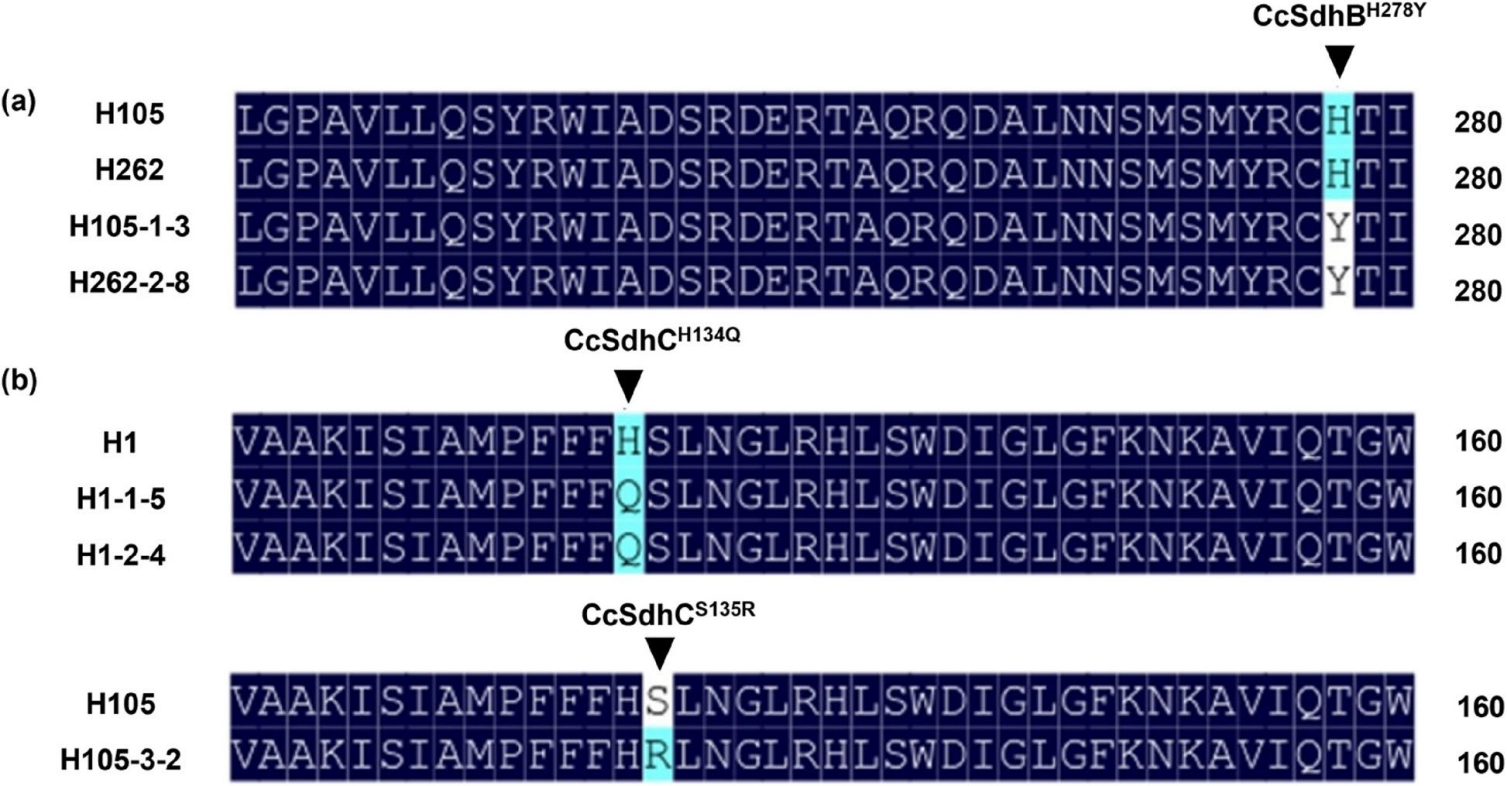
Sequence alignment of amino acid sequences of (**a**) CcSdhB and (**b**) CcSdhC from cyclobutrifluram-resistant mutants of *Corynespora cassiicola* and their parental isolates

The sequencing of *CcSdh* genes from cyclobutrifluram-resistant mutants obtained through fungicide adaptation revealed three distinct point mutations: CcSdhB^H278Y^ in mutants H105-1-3 and H262-2-8, CcSdhC^H134Q^ in mutants H1-1-5 and H1-2-4 as well as CcSdhC^S135R^ in mutant H105-3-2 (Fig. 4 and Table S7). A summary of these mutations and their associated fungicide sensitivity profiles is presented in Table 2. Furthermore, the following seven point mutations were identified in field-collected resistant isolates: CcSdhB^H278Y^, CcSdhB^I280V^, CcSdhC^S73P^, CcSdhC^N75S^, CcSdhC^H134R^, CcSdhD^D121E^ and CcSdhD^G135V^ (Table S7).

### Detection of cyclobutrifluram-resistant *C. cassiicola* isolates by allele-specific PCR (AS-PCR)

AS-PCR primers were designed based on the identified point mutations, and a systematic screening process was performed to determine the most effective primer sets and optimal annealing temperatures. Among the 253 *C. cassiicola* isolates tested, 187 carried at least one of the known resistance-associated mutations (Table S8). Notably, while several isolates harbored single-point mutations in the *CcSdh* genes, others exhibited double mutations as follows: isolate #104–2: CcSdhB^H278Y^ and CcSdhC^H134R^; isolate H199: CcSdhB^H278Y^ and CcSdhC^S73P^; isolates H47 and H50: CcSdhC^N75S^ and CcSdhB^I280V^; isolates H130 and H265: CcSdhC^S73P^ and CcSdhC^N75S^; isolate H222: CcSdhD^D121E^ and CcSdhB^I280V^; isolate H223: CcSdhC^H134R^ and CcSdhB^I280V^; isolate H255: CcSdhC^H134R^ and CcSdhD^G135V^; and isolate H170: CcSdhD^D121E^ and CcSdhC^S73P^.

### Effects of mutation type on the sensitivity to different SDHIs

Isolates carrying different point mutations exhibited varying levels of resistance to SDHI fungicides (Table 4). For instance, isolates harboring the CcSdhB^H278Y^ mutation remained sensitive to fluopyram and isofetamid, but showed low resistance to pydiflumetofen, moderate resistance to boscalid, fluxapyroxad and isopyrazam as well as high resistance to cyclobutrifluram, penflufen and thifluzamide; isolates with the CcSdhB^I280V^ mutation exhibited moderate resistance to thifluzamide, while showing low resistance to other SDHIs; those carrying the CcSdhC^S73P^ mutation remained sensitive to thifluzamide but displayed moderate resistance to cyclobutrifluram as well as low resistance to other SDHIs; isolates with the CcSdhC^N75S^ mutation were sensitive to boscalid and fluxapyroxad, while exhibiting low resistance to other SDHIs; those with the CcSdhC^H134R^ mutation displayed low resistance to fluopyram and isofetamid but high resistance to other SDHIs; isolates containing the CcSdhD^D121E^ mutation remained sensitive to fluopyram but showed moderate resistance to cyclobutrifluram and thifluzamide as well as low resistance to other SDHIs; isolates with the CcSdhD^G135V^ mutation showed high resistance to cyclobutrifluram and thifluzamide but low resistance to other SDHIs (Table 4).

**Table 4:** The EC_50_ of 9 SDHI fungicides against 32 *Corynespora cassiicola* isolates and the amino acid substitutions in CcSdh proteins

Isolates	Amino acid substitution	EC_50_ (µg/mL)								
		Cyclobutrifluram	Boscalid	Fluopyram	Fluxapyroxad	Pydiflumetofen	Isopyrazam	Penflufen	Isofetamid	Thifluzamide
04–1	—^*^	0.002	0.42	0.25	0.07	0.006	0.01	0.14	0.07	0.14
H257	—	0.011	0.19	0.17	0.01	0.007	0.02	0.13	0.11	0.05
H262	—	0.006	0.20	0.38	0.02	0.008	0.05	0.24	0.13	0.06
H85	CcSdhC^H134R^	3.12 (495.63)^#^	13.52 (50.07)	3.19 (11.96)	4.38 (131.40)	0.92 (131.43)	21.01 (787.88)	> 100.00 (> 588.24)	0.51 (4.94)	> 100.00 (> 1200.00)
H92	CcSdhC^H134R^	4.41 (696.32)	17.60 (65.19)	1.52 (5.70)	8.70 (261.00)	1.38 (197.14)	65.75 (2465.63)	> 100.00 (> 588.24)	0.55 (5.32)	> 100.00 (> 1200.00)
H268	CcSdhB^H278Y^	1.66 (262.11)	18.49 (68.48)	0.13 (0.49)	0.82 (201.00)	0.05 (7.14)	0.90 (33.75)	41.62 (244.82)	0.04 (0.39)	> 100.00 (> 1200.00)
H274	CcSdhB^H278Y^	0.88 (138.95)	15.03 (55.67)	0.13 (0.49)	0.76 (22.80)	0.09 (12.86)	0.37 (13.88)	> 100.00 (> 588.24)	0.19 (1.84)	> 100.00 (> 1200.00)
#47–3	CcSdhB^I280V^	0.08 (12.63)	0.27 (1.00)	2.20 (8.25)	0.10 (3.00)	0.01 (1.43)	0.05 (1.88)	0.76 (4.47)	0.13 (1.26)	3.56 (42.72)
#47–4	CcSdhB^I280V^	0.02 (3.16)	1.05 (3.89)	10.79 (40.46)	0.12 (3.60)	0.05 (7.14)	0.24 (9.00)	4.81 (28.29)	0.39 (3.77)	18.33 (219.96)
H148	CcSdhB^I280V^	0.03 (4.74)	0.79 (2.93)	1.64 (6.15)	0.22 (6.60)	0.05 (7.14)	0.24 (9.00)	15.93 (93.71)	0.42 (4.06)	28.65 (343.80)
H83	CcSdhC^S73P^	0.57 (90.00)	1.17 (4.43)	2.57 (9.64)	1.06 (31.80)	0.07 (10.00)	0.07 (2.63)	0.34 (2.00)	1.49 (14.42)	0.03 (0.36)
H106	CcSdhC^S73P^	0.27 (42.63)	2.19 (8.11)	4.20 (15.75)	0.51 (15.30)	0.15 (21.43)	0.09 (3.38)	2.66 (15.65)	1.60 (15.48)	0.15 (1.80)
H136	CcSdhC^S73P^	0.36 (56.84)	1.24 (4.59)	1.25 (4.69)	0.81 (24.30)	0.12 (17.14)	0.08 (3.00)	2.94 (17.29)	0.59 (5.71)	0.08 (0.96)
H240	CcSdhC^S73P^	0.54 (85.26)	4.27 (15.81)	6.16 (23.10)	0.51 (15.30)	0.11 (15.71)	0.09 (3.38)	0.69 (4.06)	3.57 (34.55)	0.02 (0.24)
H248	CcSdhC^S73P^	0.53 (83.68)	3.91 (14.48)	4.11 (15.41)	0.32 (9.60)	0.04 (5.71)	0.07 (2.63)	1.94 (11.41)	3.32 (32.13)	0.03 (0.36)
#41–2	CcSdhC^S73P^	0.32 (50.53)	1.71 (6.33)	3.61 (13.54)	0.60 (18.00)	0.07 (10.00)	0.06 (2.25)	1.90 (11.18)	2.49 (24.10)	0.04 (0.48)
H4	CcSdhC^N75S^	0.13 (20.53)	0.24 (0.89)	0.45 (1.69)	0.18 (5.40)	0.10 (14.29)	0.37 (13.88)	1.39 (8.18)	1.19 (11.52)	1.05 (12.60)
#41–3	CcSdhC^N75S^	0.14 (22.11)	0.23 (0.85)	1.43 (5.36)	0.17 (5.10)	0.04 (5.71)	0.19 (7.13)	1.80 (10.59)	0.31 (3.00)	1.09 (13.08)
H65	CcSdhD^D121E^	0.77 (121.58)	0.44 (1.63)	1.19 (4.46)	0.23 (6.90)	0.06 (8.57)	0.20 (7.50)	0.80 (4.71)	0.72 (6.97)	2.68 (32.16)
H70	CcSdhD^D121E^	0.66 (104.21)	0.37 (1.37)	0.32 (1.20)	0.25 (7.50)	0.06 (8.57)	0.13 (4.88)	0.35 (2.06)	0.44 (4.26)	1.39 (16.68)
H111	CcSdhD^G135V^	1.18 (186.32)	1.50 (5.56)	0.69 (2.59)	0.33 (9.90)	0.10 (14.29)	0.22 (8.25)	1.82 (10.71)	0.94 (9.10)	4.97 (59.64)
H112	CcSdhD^G135V^	1.87 (295.26)	2.06 (7.63)	2.52 (9.45)	0.44 (13.20)	0.11 (15.71)	0.44 (16.50)	1.94 (11.41)	1.50 (14.52)	11.03 (132.36)
H130	CcSdhC^S73P^ + CcSdhC^N75S^	0.86 (135.79)	0.89 (3.30)	1.05 (3.94)	0.32 (9.60)	0.03 (4.29)	0.02 (0.75)	0.18 (1.06)	12.11 (117.19)	0.04 (0.48)
H265	CcSdhC^S73P^ + CcSdhC^N75S^	1.74 (274.74)	3.80 (14.07)	11.00 (41.25)	1.38 (41.40)	0.75 (107.14)	0.18 (6.75)	1.22 (7.18)	25.37 (245.52)	0.22 (2.64)
#104–2	CcSdhB^H278Y^ + CcSdhC^H134R^	1.75 (276.32)	25.26 (93.56)	0.16 (0.60)	0.66 (19.80)	0.81 (115.71)	0.86 (32.25)	> 100.00 (> 588.24)	0.05 (0.48)	> 100.00 (> 1200.00)
H199	CcSdhB^H278Y^ + CcSdhC^S73P^	3.27 (516.32)	39.32 (145.63	0.38 (1.43)	5.10 (153.00)	0.89 (127.14)	3.46 (129.75)	> 100.00 (> 588.24)	0.11 (1.06)	> 100.00 (> 1200.00)
H47	CcSdhC^N75S^ + CcSdhB^I280V^	0.12 (18.95)	0.35 (1.30)	1.08 (4.05)	0.12 (3.60)	0.04 (5.71)	0.14 (5.25)	0.72 (4.24)	1.19 (11.52)	6.43 (77.16)
H50	CcSdhC^N75S^ + CcSdhB^I280V^	0.06 (9.47)	0.16 (0.59)	2.12 (7.95)	0.06 (1.80)	0.01 (1.43)	0.05 (1.88)	0.29 (1.71)	0.82 (7.94)	4.80 (57.60)
H222	CcSdhD^D121E^ + CcSdhB^I280V^	0.02 (3.16)	0.44 (1.63)	2.09 (7.84)	0.10 (3.00)	0.02 (2.86)	0.12 (4.50)	0.77 (4.53)	1.34 (12.97)	7.60 (91.20)
H223	CcSdhC^H134R^ + CcSdhB^I280V^	0.08 (12.63)	0.25 (0.93)	28.56 (107.10)	0.12 (3.60)	0.01 (1.43)	0.10 (3.75)	1.60 (9.41)	1.09 (10.55)	18.96 (227.52)
H255	CcSdhC^H134R^ + CcSdhD^G135V^	0.21 (33.16)	0.63 (2.33)	1.03 (3.86)	0.21 (6.30)	0.05 (7.14)	0.34 (12.75)	1.26 (7.41)	0.40 (3.87)	10.26 (123.12)
H170	CcSdhD^D121E^ + CcSdhC^S73P^	0.45 (71.05)	0.96 (3.56)	0.88 (3.30)	0.31 (9.30)	0.04 (5.71)	0.04 (1.50)	0.18 (1.06)	5.48 (53.03)	0.04 (0.48)

^*^ Isolate does not contain a mutation

^#^ RF (Resistance factor) to SDHI fungicides. RF = the ratio of the EC_50_ of the mutated isolates to the mean EC_50_ of three non-mutated isolates

Furthermore, isolates harboring double mutations displayed diverse resistance profiles to SDHIs. Notably, most exhibited either increased or unchanged resistance compared with single-mutation isolates. For example, isolates with the CcSdhC^S73P^ mutation had EC_50_ values ranging from 0.30 to 0.60 µg/mL for cyclobutrifluram, while those with the CcSdhC^N75S^ mutation showed EC_50_ values of 0.13−0.14 µg/mL. In contrast, isolates containing both mutations (CcSdhC^S73P^ and CcSdhC^N75S^) had elevated EC_50_ values of 0.80−1.74 µg/mL, indicating low to moderate resistance to cyclobutrifluram. Interestingly, in certain cases, the presence of a second mutation also reduced overall resistance. For instance, when CcSdhB^H278Y^ was included in the double mutation, the isolates remained sensitive to fluopyram and isofetamid, despite other mutations conferring resistance. Similarly, when CcSdhC^S73P^ was part of a double mutation, the isolates maintained sensitivity to thifluzamide, regardless of resistance conferred by additional mutations (Table 4).

### Cross-resistance between cyclobutrifluram and other SDHIs

Specific mutations in the *CcSdh* genes influence cross-resistance patterns between cyclobutrifluram and other SDHI fungicides. Specifically, when the CcSdhB^H278Y^ mutation was present, cyclobutrifluram did not exhibit cross-resistance with fluopyram or isofetamid, although positive cross-resistance was detected with other SDHIs. In the case of the CcSdhC^S73P^ mutation, cyclobutrifluram exhibited negative cross-resistance with thifluzamide, while showing positive cross-resistance with the other SDHIs. Additionally, for the CcSdhC^N75S^ mutation, cyclobutrifluram exhibited no cross-resistance with boscalid or fluxapyroxad but showed positive cross-resistance with the remaining SDHIs. Similarly, isolates with the CcSdhD^D121E^ mutation showed no cross-resistance between cyclobutrifluram and fluopyram, yet demonstrated positive cross-resistance with other SDHIs. Finally, when the CcSdhB^I280V^, CcSdhC^H134R^ or CcSdhD^G135V^ mutations were present, cyclobutrifluram exhibited positive cross-resistance with all eight tested SDHI fungicides. The corresponding *P* and *ρ* values are presented in Table 5.

**Table 5:** Cross-resistance between cyclobutrifluram and other SDHI fungicides

Fungicide	Cross resistance^*^						
	CcSdhB^H278Y^	CcSdhB^I280V^	CcSdhC^S73P^	CcSdhC^N75S^	CcSdhC^H134R^	CcSdhD^D121E^	CcSdhD^G135V^
Boscalid	*P* < 0.05; *ρ* = 0.71 *df* = 10	*P* < 0.05; *ρ* = 0.42; *df* = 6	*P* < 0.05; *ρ* = 0.71 *df* = 10	***P* = 0.90; *ρ* = −0.024; *df* = 6**	*P* < 0.05; *ρ* = 0.56; *df* = 7	*P* < 0.05; *ρ* = 0.44; *df* = 7	*P* < 0.05; *ρ* = 0.61; *df* = 7
Fluopyram	***P* = 0.09; ρ = −0.34; *df* = 8**	*P* < 0.05; *ρ* = 0.63; *df* = 7	*P* < 0.05; *ρ* = 0.73; *df* = 10	*P* < 0.05; *ρ* = 0.54; *df* = 6	*P* < 0.05; *ρ* = 0.63; *df* = 7	***P* = 0.55; *ρ* = 0.12; *df* = 7**	*P* < 0.05; *ρ* = 0.66; *df* = 7
Fluxapyroxad	*P* < 0.05; *ρ* = 0.68; *df* = 10	*P* < 0.05; *ρ* = 0.48; *df* = 7	*P* < 0.05; *ρ* = 0.76; *df* = 10	***P* = 0.14; *ρ* = 0.34; *df* = 6**	*P* < 0.05; *ρ* = 0.61; *df* = 7	*P* < 0.05; *ρ* = 0.61; *df* = 7	*P* < 0.05; *ρ* = 0.63; *df* = 7
Pydiflumetofen	*P* < 0.05; *ρ* = 0.72; *df* = 10	*P* < 0.05; *ρ* = 0.75; *df* = 7	*P* < 0.05; *ρ* = 0.75; *df* = 10	*P* < 0.05; *ρ* = 0.79; *df* = 6	*P* < 0.05; *ρ* = 0.87; *df* = 7	*P* < 0.05; *ρ* = 0.82; *df* = 7	*P* < 0.05; *ρ* = 0.87; *df* = 7
Isopyrazam	*P* < 0.05; *ρ* = 0.82; *df* = 10	*P* < 0.05; *ρ* = 0.59; *df* = 7	*P* < 0.05; *ρ* = 0.67; *df* = 10	*P* < 0.05; *ρ* = 0.62; *df* = 6	*P* < 0.05; *ρ* = 0.75; *df* = 7	*P* < 0.05; *ρ* = 0.73; *df* = 7	*P* < 0.05; *ρ* = 0.75; *df* = 7
Penflufen	*P* < 0.05; *ρ* = 0.83; *df* = 10	*P* < 0.05; *ρ* = 0.68; *df* = 7	*P* < 0.05; *ρ* = 0.68; *df* = 10	*P* < 0.05; *ρ* = 0.63; *df* = 6	*P* < 0.05; *ρ* = 0.71; *df* = 7	*P* < 0.05; *ρ* = 0.42; *df* = 7	*P* < 0.05; *ρ* = 0.73; *df* = 7
Isofetamid	***P* = 0.07; *ρ* = −0.38; *df* = 10**	*P* < 0.05; *ρ* = 0.72; *df* = 7	*P* < 0.05; *ρ* = 0.85; *df* = 10	*P* < 0.05; *ρ* = 0.81; *df* = 6	*P* < 0.05; *ρ* = 0.82; *df* = 7	*P* < 0.05; *ρ* = 0.88; *df* = 7	*P* < 0.05; *ρ* = 0.88; *df* = 7
Thifluzamide	*P* < 0.05; *ρ* = 0.80; *df* = 9	*P* < 0.05; *ρ* = 0.56; *df* = 6	*P* < 0.05; *ρ* = −0.55; *df* = 9	*P* < 0.05; *ρ* = 0.45; *df* = 5	*P* < 0.05; *ρ* = 0.6; *df* = 6	*P* < 0.05; *ρ* = 0.56; *df* = 6	*P* < 0.05; *ρ* = 0.56; *df* = 6

^*^ Values in bold indicate that cyclobutrifluram has no cross-resistance with the tested fungicide; The degrees of freedom (*df*) in cross-resistance analysis (Spearman’s rank correlation coefficient analysis) are defined by *df* = *n* − 2, where *n* is the number of strains tested including both wild-type isolates and resistant mutants

## Discussion

SDHIs have wide applications in the management of crop diseases, especially with their potential to disrupt the respiratory activity of phytopathogenic fungi through inhibition of succinate dehydrogenase (Avenot and Michailides 2010). Carboxin, the first commercial SDHI, was introduced for controlling cereal diseases caused by Basidiomycete fungi and was primarily used as a seed or soil treatment (Ulrich and Mathre 1972). Since 2003, newer generations of SDHIs, characterized by a broader antifungal spectrum as well as enhanced activity against Basidiomycetes, Ascomycetes and anamorphic fungi, has been developed (Avenot and Michailides 2010). The present study investigated the bioactivity of the novel SDHI fungicide cyclobutrifluram which is known to exhibit strong inhibitory effects on various plant pathogens. This compound tends to be particularly effective against anamorphic fungi but demonstrates limited activity against members of the phyla Basidiomycota, Oomycota and Ascomycota, with the notable exceptions of *Fusarium* spp., *Cochliobolus* and *Mycosphaerella*. Indeed, unlike earlier SDHIs, cyclobutrifluram has shown high efficacy against 11 species of *Fusarium* (Li et al. 2024), despite its otherwise limited activity against Basidiomycota. According to the FRAC, SDHIs can be classified based on their chemical structures, which include pyrazine-carboxamides, furan-carboxamides, thiazole-carboxamides, pyridine-carboxamides and phenyl-benzamides (https://www.frac.info). The unique properties of the cyclobutrifluram pyridine moiety as well as its specific interactions with succinate dehydrogenase may contribute to its distinct activity spectrum. Therefore, a thorough understanding of the antifungal spectrum of cyclobutrifluram is essential for guiding its registration and field application.

Cucumber target spot has recently emerged as a highly destructive disease affecting cucumbers, leading to significant economic losses (Peng et al. 2024a), and its management relies heavily on chemical fungicides. However, the increasing incidence of fungicide resistance highlights the urgent need for novel and effective control agents. In this context, cyclobutrifluram, with its demonstrated activity against both nematodes and a broad range of plant fungal pathogens (http://www.pesticidenews.cn), presents itself as a promising candidate for controlling cucumber target spot. In this study, 171 *C. cassiicola* isolates were selected to assess their sensitivity to cyclobutrifluram, and the results revealed a wide range of EC_50_ values, from 0.0071 to 58.70 μg/mL, which reflected the presence of field-resistant isolates. This substantial variability also underscores the importance of resistance monitoring and indicates the need for appropriate resistance management strategies when deploying cyclobutrifluram in the field.

Five cyclobutrifluram-resistant mutants were successfully generated through fungicide adaptation, and their CFIs were thoroughly analyzed. Overall, the resistant mutants showed significantly lower CFIs compared with their respective parental isolates in all but one case, suggesting that resistance is generally associated with a fitness cost. Furthermore, disease control assays demonstrated that cyclobutrifluram was less effective against cucumber target spot caused by resistant mutants than against that caused by the parental isolates. These findings indicated that the emergence of resistance can compromise disease control, thus underscoring the need for continuous surveillance. Moreover, cyclobutrifluram did not show cross-resistance with other commonly used fungicides such as florylpicoxamid, pyraclostrobin, prochloraz and propineb. In summary, the observed variability in sensitivity and emergence of resistant isolates suggested that the resistance risk is moderate to high.

In this study, no overexpression of *CcSdhA*_*1*_, *CcSdhA*_*2*_, *CcSdhB*, *CcSdhC*, or *CcSdhD* genes was observed, but the following nine distinct point mutations were identified in cyclobutrifluram-resistant mutants or parental isolates: CcSdhB^H278Y^, CcSdhB^I280V^, CcSdhC^S73P^, CcSdhC^N75S^, CcSdhC^H134R^, CcSdhC^H134Q^, CcSdhC^S135R^, CcSdhD^D121E^ and CcSdhD^G135V^. These mutations are associated with resistance to SDHI fungicides and have been previously reported in other plant pathogens (Amiri et al. 2014; Avenot et al. 2009; Avenot and Michailides 2010; Malandrakis et al. 2017; Pearce et al. 2019; Peng et al. 2024b, 2025; Rehfus et al. 2018; Sun et al. 2017). Further analysis revealed that isolates harboring these mutations could exhibit varying degrees of resistance to cyclobutrifluram and other SDHI fungicides. Notably, isolates with the CcSdhB^H278Y^ or CcSdhC^S73P^ mutations displayed differential resistance to most SDHIs while remaining sensitive to fluopyram, isofetamid or thifluzamide. Fluopyram and isofetamid belong to the stretched heterocycle amide SDHIs (SHA-SDHIs), characterized by an aliphatic C−C linker rather than an aromatic ring at the amide moiety (Steinhauer et al. 2019). Previous research suggested that *Podosphaera xanthii* and *C. cassiicola* mutants with the SdhB^H278Y^ mutation could exhibit resistance to boscalid and penthiopyrad while remaining sensitive to fluopyram (Ishii et al. 2011). The histidine residue at codon 278 of the SdhB subunit is a highly conserved component of the [3Fe-4S] iron-sulfur cluster (Alzohairy et al. 2023). This residue forms a hydrogen bond with the O3-methoxy group of ubiquinone and plays a crucial role in SDHI binding (Leroux et al. 2010). However, substitution of histidine with tyrosine (H278Y) disrupts this interaction, leading to resistance to certain SDHIs, such as boscalid and cyclobutrifluram, while paradoxically enhancing sensitivity to SHA-SDHIs such as fluopyram and isofetamid. This phenomenon may result from structural changes that alter compatibility with different fungicides. Thus, it is likely that fluopyram and isofetamid may better accommodate the altered binding site geometry induced by the tyrosine substitution. Molecular docking, a rapidly advancing computational technique, allows the simulation of binding conformations and prediction of binding affinities between small molecules and biomacromolecules (Usman et al. 2025). Future studies could apply this method to analyze the binding interactions between SDHIs (e.g., cyclobutrifluram, fluopyram and isofetamid) and mutated or wild-type CcSdhB/C/D proteins, thereby elucidating the structural basis for the observed sensitivity differences.

The strategic use of fungicides with distinct modes of action, either through rotation or combination, can significantly delay the development of SDHI resistance (Li et al. 2021; Ma et al. 2020). In particular, given that cyclobutrifluram did not exhibit cross-resistance with propineb, pyraclostrobin, prochloraz or florylpicoxamid, it could be used in combination or alternately with these fungicides to mitigate resistance risks. Furthermore, *C. cassiicola* isolates carrying specific Sdh mutations exhibited differential sensitivity to other SDHIs. For instance, isolates with SdhB^H278Y^, SdhC^S73P^ or SdhC^N75S^ mutations remained sensitive to fluopyram, isofetamid, thifluzamide and boscalid. Therefore, the selection of appropriate SDHIs based on detected mutations (e.g., using AS-PCR) offers a rational approach for managing resistant populations (e.g., fluopyram or isofetamid for SdhB^H278Y^, boscalid for SdhC^N75S^ and thifluzamide for SdhC^S73P^ mutations). Additionally, when other mutations are predominant, integrated strategies that combine fungicides with distinct modes of action are recommended. However, recent studies have reported rising resistance to QoIs (Zhou et al. 2024; Zhu et al. 2024), DMIs (Deng et al. 2024) and other fungicide classes (Mao et al. 2022). Therefore, continuous monitoring of fungicide resistance in *C. cassiicola* and elucidation of its underlying molecular mechanisms are critical before implementing alternation or combination strategies to sustainably manage cyclobutrifluram resistance in the field.

The emergence of cyclobutrifluram resistance in *C. cassiicola* in field populations may be partially attributed to cross-resistance with other SDHI fungicides. Indeed, since June 2013, SDHIs, such as fluopyram, fluxapyroxad and pydiflumetofen, have been registered in China for controlling cucumber target spot. Additionally, the approval of other SDHIs for managing different cucumber diseases may have further contributed to the development of cyclobutrifluram resistance. For example, boscalid, isofetamid and isopyrazam have been in use since November 2005 to control cucumber powdery mildew and cucumber grey mold. The prolonged and frequent application of these fungicides likely exerted selective pressure, gradually leading to the emergence of resistant strains and subpopulations in the field.

## Conclusion

The current research demonstrated that cyclobutrifluram was effective against anamorphic fungi and selected ascomycetes. However, the resistance risk in *C. cassiicola* was classified as moderate to high, primarily driven by mutations in the *CcSdh* genes. Cross-resistance between cyclobutrifluram and other SDHI fungicides were also found to be mutation-dependent, varying with specific alterations in the Sdh proteins. Overall, these findings offer practical guidance for the control of *C. cassiicola* and for the management of cyclobutrifluram resistance in agricultural systems.

## Materials and methods

### Fungicides

Cyclobutrifluram (80% active ingredient [a.i.]), cyclobutrifluram suspension concentrate (SC, 10 g/L), pydiflumetofen (98% a.i.) and isopyrazam (97% a.i.) were provided by Syngenta Biotechnology Co., Ltd. (Shanghai). Florylpicoxamid (100% a.i.) was obtained from Corteva Agriscience Co., Ltd. (Shanghai), while Bayer Crop Science (Shanghai) provided penflufen (95.6% a.i.) and fluopyram (96% a.i.). Fluxapyroxad (98% a.i.) and pyraclostrobin (98.6% a.i.) were obtained from BASF China Co., Ltd. (Shanghai). Additional fungicides were purchased from commercial sources: isofetamid (98.4% a.i.; Shenyang Sciencreat Chemicals Co., Ltd.), thifluzamide (98.4% a.i.; Nutrichem Company Limited), propineb (100% a.i.; Aladdin), prochloraz (100% a.i.; Jiangsu Huifeng Bio Agriculture Co., Ltd.) and boscalid (98.6% a.i.; Beijing Mingdelida Agricultural Technology Co., Ltd.). Stock solutions (10^5^ μg/mL) of all fungicides were prepared in dimethyl sulfoxide (DMSO) and kept in the dark at 4℃ until needed.

### Plant pathogens and culture media

The plant pathogens, culture media and incubation temperatures used for evaluating the antifungal spectrum of cyclobutrifluram as well as *C. cassiicola*’s sensitivity to the fungicide are listed in Tables S1 and S2. Potato dextrose agar (PDA; 15 g/L agar, 18 g/L glucose and 200 g/L potato) and yeast bacterial peptone agar (YBA; 15 g/L agar, 10 g/L bacterial peptone, 10 g/L yeast extract and 20 g/L sodium acetate), both prepared in deionized water, were used to assess the sensitivity of the plant pathogens to cyclobutrifluram. PDA was also used for isolating, subculturing and assessing the fitness of *C. cassiicola*, with YBA employed to determine *C. cassiicola*’s sensitivity to SDHI and other fungicides through cross-resistance analysis. Additionally, water agar (WA; 15 g agar in 1 L deionized water) was used for spore germination, while for total RNA extraction from *C. cassiicola*, yeast bacterial peptone broth (YBB; YBA without 15 g agar) was used. All media were sterilized at 121℃ for 20 min (Miao et al. 2020).

### Antifungal spectrum of cyclobutrifluram

To evaluate the antifungal spectrum of cyclobutrifluram, 39 plant pathogenic fungi (Table S1) were tested for their sensitivity. For this purpose, fresh, 5-mm wide mycelial plugs taken from the margins of actively growing colonies were transferred to agar plates that had been supplemented with 10 or 100 µg/mL of cyclobutrifluram, with each treatment consisting of three replicates. Plates containing 0.1% DMSO were also included as untreated controls. All cultures were then incubated at the appropriate optimal temperature in the dark until the control colonies reached a diameter of approximately 7 cm. Colony diameters were subsequently measured perpendicularly, with the percentage inhibition of mycelial growth eventually determined as follows: (colony diameter of control − colony diameter of treatment)/(colony diameter of control – 5 mm) × 100. For fungal species found to be sensitive to cyclobutrifluram, dose–response assays were conducted using a range of concentrations (0, 0.003, 0.006, 0.01, 0.02, 0.05, 0.2, 1 and 2 µg/mL) to determine the median effective concentration (EC_50_). In this case, EC_50_ values were determined using probit regression analysis, correlating percent inhibition with the logarithm of fungicide concentration, as previously described (Pang et al. 2013).

### *C. cassiicola*’s sensitivity to cyclobutrifluram

A total of 171 *C. cassiicola* isolates (Table S2) were evaluated for sensitivity to cyclobutrifluram using the mycelial growth rate method. Five-millimeter-wide mycelial plugs taken from the margins of four-day-old colonies were placed on YBA plates that had been supplemented with different concentrations of cyclobutrifluram (Table S3). Following a 5-day incubation at 25℃, colony diameters were measured in two perpendicular directions before determining EC_50_ values as described above. Three replicates were included per treatment, with the whole experiment also repeated twice.

### Selection of *C. cassiicola* mutants exhibiting cyclobutrifluram resistance

Ten wild-type *C. cassiicola* isolates (H1, H12, WF-12, TA-2, H105, H96, H257, #41–3, #73–1 and H262), collected from different parts of China, were selected for cyclobutrifluram adaptation on YBA medium. Five-millimeter-wide mycelial plugs, excised from seven-day-old colonies, were then placed on YBA plates supplemented with 1 μg/mL of cyclobutrifluram. Preliminary tests confirmed that this concentration fully inhibited the growth of sensitive isolates, with no growth observed at 0.5 μg/mL. After 15−20 days of incubation at 25℃ in the dark, colonies exhibiting growth (i.e., putative mutants) were transferred to fungicide-free YBA plates, followed by subculturing onto YBA containing 0.5 µg/mL cyclobutrifluram. Mutants that still showed normal growth were further transferred to plates containing increasingly higher levels of cyclobutrifluram (0.5, 1, 2, 5 and 10 µg/mL). Sensitivity to cyclobutrifluram was eventually determined for each mutant based on the resistance factor (RF), calculated as the ratio of the EC_50_ value of the resistant mutant to that of its corresponding parental isolate.

### Characterization of *C. cassiicola* mutants exhibiting cyclobutrifluram resistance

#### Resistance stability

To assess resistance stability, five resistant *C. cassiicola* mutants (H1-1-5, H1-2-4, H105-1-3, H105-3-2 and H262-2-8) and their sensitive parental isolates (H1, H105 and H262) were successively subcultured onto fungicide-free PDA plates ten times (Table 3). The sensitivity of the first and tenth generations was then determined using the mycelial growth rate method, while resistance stability was evaluated by calculating the RF and the factor of sensitivity change (FSC).

### Sensitivity to temperature and mycelial growth rate

To compare growth characteristics under different temperatures, 5 mm mycelial plugs, taken from four-day-old colonies of resistant mutants and parental isolates, were placed on fungicide-free PDA plates. Following a 7-day incubation in the dark at 4, 13, 22, 25, 28, 30 and 37℃, the diameters of the resulting colonies were measured perpendicularly. There were three replicates for each temperature treatment, with the experiment also repeated three times.

### Conidial production and germination rate

To assess conidiation, isolates were grown for 10 days on PDA, with incubation performed in the dark at 25°C. Conidia were then harvested by rinsing the plates with sterile water, followed by filtration through three layers of gauze, with conidial counts subsequently determined using a hemocytometer under a microscope. Conidial suspensions were then centrifuged at 4000 rpm for 5 min before being re-suspended and adjusted to 1 × 10^5^ conidia/mL. For germination assays, 50 µL aliquots of these suspensions were evenly spread on WA plates and after incubating for 8 h in the dark at 25°C (Dixon et al. 2009), germination rates were assessed by randomly counting 100 conidia per plate under a microscope. For each strain, triplicate tests were performed, with the experiment also repeated three times.

### Pathogenicity and control efficacy

Cucumber plants (cultivar: Jinyan No. 4) were grown to the three-leaf stage in a greenhouse. Detached leaves were then inoculated with mycelial plugs from resistant mutants or their respective parental isolates (Table 3), with lesion areas subsequently measured after 3−5 days of incubation in a phytotron at 25℃ and 80% humidity. Furthermore, to evaluate the protective efficacy of cyclobutrifluram, leaves were pre-sprayed with 50 µg/mL cyclobutrifluram 24 h before inoculation with *C. cassiicola*. In this case, controls were also included by treating leaves with water containing an equal volume of DMSO. Lesion areas were then measured 3−5 days post inoculation before calculating the control efficacy with the following equation: (lesion area of control – lesion area of treatment)/(lesion area of control) × 100. Each treatment contained 6−8 leaves, with three replicates per isolate. The experiment was also repeated thrice.

### Cross-resistance assessment

To assess potential cross-resistance, the sensitivities of 23 *C. cassiicola* isolates (including resistant mutants and sensitive isolates) were evaluated against cyclobutrifluram, florylpicoxamid, pyraclostrobin, prochloraz and propineb. Fresh 5-mm-wide mycelial plugs, taken from the colony margins, were placed onto agar plates containing each fungicide at concentrations listed in Table S3. Additionally, to examine cross-resistance among SDHIs, resistant isolates with known mutations were tested for sensitivity to boscalid, fluopyram, fluxapyroxad, pydiflumetofen, isopyrazam, penflufen, isofetamid and thifluzamide. The resistance of the isolates were subsequently categorized as follows based on RF values: low (10 < RF < 50), moderate (50 < RF < 100) and high (RF > 100) (Li et al. 2021). Finally, Spearman’s rank correlation coefficient was used for analyzing cross-resistance between cyclobutrifluram and the tested fungicides (Gao et al. 2022). These experiments were repeated three times.

### Determination of *CcSdh* genes’ expression levels

Five 5-mm-wide mycelial plugs from cyclobutrifluram-resistant mutants and their corresponding sensitive parental isolates (Table 3) were inoculated into flasks containing 100 mL YBB. Following a 2-day incubation in the dark at 25℃ and 175 rpm, cyclobutrifluram was added to each flask at a concentration equivalent to the EC_50_ value specific to each strain. For the control, an equal volume of DMSO was used, and mycelia were subsequently harvested following an additional two-day incubation. The EASY Universal Plant RNA Rapid Extraction Kit/DNase I (Genenode Biotech Co. Ltd.) was used for total RNA extraction prior to cDNA synthesis with the *EasyScript* One-Step gDNA Removal and cDNA Synthesis SuperMix Kit (TransGen Biotech Co. Ltd.). This was followed by real-time PCR, conducted using the primers listed in Table S4 along with the TB Green Premix Ex Taq II (Tli RNaseH Plus) Kit (Takara Bio Inc.). The 2^−ΔΔCt^ method was eventually used for calculating the relative expression level of the *CcSdh* genes, with the *CcActin* gene serving as the internal reference. Each experiment was performed in triplicate.

### Cloning and sequencing of *CcSdh* genes

Mycelia were obtained by culturing resistant mutants along with their respective sensitive parental isolates (Table 3). The fungi were grown for 7 days on PDA plates, with incubation performed in the dark at 25℃. Genomic DNA, extracted from 30 mg of mycelium using the 3% CTAB method (Nazhad and Solouki 2008), was then used for amplifying full-length sequences of the *CcSdh* genes, with the amplification process achieved using the primers listed in Table S4 along with 2 × M5 Taq PCR Mix with Blue Dye (Mei5 Biotechnology Co., Ltd.). The resulting products were sent to Beijing Tsingke Biological Technology Co., Ltd. for sequencing, with sequence analysis performed using SnapGene software.

### Allele-specific PCR for detecting mutations in *CcSdh* genes

Mutations in the *CcSdhB*, *CcSdhC* and *CcSdhD* genes were identified in both resistant and sensitive parental isolates. Based on these mutations, AS-PCR primers (Table S5) were designed to detect nucleotide substitutions in those genes. Additionally, the 3'-end of these primers were tailored to match the resistant allele at the mutation site, with primer specificity also enhanced by introducing a base mismatch at the second base from that end. PCR was performed based on a standardized protocol using DNA templates from both resistant and sensitive isolates, with annealing temperatures ranging from 58 to 74℃. The amplified fragments were then analyzed via electrophoresis using 1% agarose gel to identify primers with optimal specificity and annealing conditions. Once optimized, genomic DNA from 253 *C. cassiicola* isolates was amplified using the selected allele-specific primers. The resulting DNA fragments were subsequently analyzed via 1% agarose gel electrophoresis to detect the presence of target mutations in *CcSdh* genes. Each experiment was repeated three times.

### Statistical analyses

The data were analyzed using the Data Processing System v.6.55 software (Hangzhou RuiFeng Information Technology Co., Ltd., Hangzhou, China). Mean differences were evaluated using Tukey’s honest significant difference (HSD) test at a significance level of *P* ≤ 0.05.

## Supplementary Information

Supplementary Material 1: Table S1 Pathogens used in this study. Table S2 Information on *Corynespora cassiicola* isolates collected from 2014 to 2019. Table S3 Concentrations used to determine the sensitivity to different fungicides of wild-type isolates and cyclobutrifluram-resistant mutants of *Corynespora cassiicola*. Table S4 Primers used to amplify and quantify the *CcSdh* genes in this study. Table S5 AS-PCR primers used to detect cyclobutrifluram-resistant isolates of *Corynespora cassiicola*. Table S6 Mycelial growth of *Corynespora cassiicola* isolates at various temperatures on PDA plates. ^*^Isolates in bold are parents of the resistant mutants; ^#^Mean ± standard error in a column followed by the same letter means there is no significant difference in ANOVA with Tukey’s HSD test at *P *< 0.05. Table S7 Information on point mutations in this study. Table S8 Detection by AS-PCR of cyclobutrifluram-resistant* Corynespora cassiicola* isolates collected from the field. ^*^253 *C. cassiicola* isolates collected from the field were used in the AS-PCR detection. Fig. S1 EC_50_ values of 171 *Corynespora cassiicola* isolates to cyclobutrifluram. Fig. S2 Expression levels of *CcSdh* genes in cyclobutrifluram-resistant mutants of *Corynespora cassiicola* and their parental isolates. Transcript levels were normalized to the expression levels of *CcSdh* genes in the sensitive isolate H1 in the absence of fungicide: (a) *CcSdhA1*, (b) *CcSdhA2*, (c) *CcSdhB*, (d) *CcSdhC*, and (e) *CcSdhD*. 

## Data Availability

Data and materials will be made available on request.

## Abbreviations

DMI: Sterol demethylation inhibitor
QoI: Quinone outside inhibitor
SDHI: Succinate dehydrogenase inhibitor
FRAC: Fungicide resistance action committee
EC_50_: Median effective concentration
RF: Resistance factor
FSC: Factor of sensitivity change
CFI: Compound fitness index
SC: Suspension concentrate
DMSO: Dimethyl sulfoxide
PDA: Potato dextrose agar
YBA: Yeast bacterial peptone agar
YBB: Yeast bacterial peptone broth
WA: Water agar
HSD: Honest significant difference
AS-PCR: Allele-specific PCR
