# Supplementary material for: Antifungal spectrum of cyclobutrifluram and multi-point mutations in CcSdh proteins confer resistance in Corynespora cassiicola
Source: Stress Biol. 2025 Sep 1;5(1):53. doi: 10.1007/s44154-025-00251-8 (PMC12399480; doi:10.1007/s44154-025-00251-8)

**Table S1**

| Phylum | Pathogen | Medium | Temperature (℃) |
| --- | --- | --- | --- |
| Basidiomycete | *Rhizoctonia cerealis* | PDA | 18 |
|  | *Rhizoctonia solani* | PDA | 25 |
|  | *Sclerotium rolfsii* | PDA | 25 |
| Anamorphic fungi | *Botrytis cinerea* | PDA | 22 |
|  | *Peyronellaea arachidicola* | PDA | 25 |
|  | *Cladosporium cucumerium* | PDA | 25 |
|  | *Alternaria brassicae* | PDA | 25 |
|  | *Alternaria solani* | PDA | 25 |
|  | *Ascochyta citrullina* | PDA | 25 |
|  | *Cercospora arachidicol* | PDA | 25 |
|  | *Corynespora cassiicola* | PDA | 25 |
|  | *Alternaria mali* | PDA | 25 |
|  | *Exserohilum turcicum* | PDA | 25 |
|  | *Bipolaris maydis* | PDA | 25 |
|  | *Macrophoma musae* | PDA | 25 |
| Ascomycetes | *Cochliobolus lunatus* | PDA | 25 |
|  | *Mycosphaerella fijiensis* | PDA | 25 |
|  | *Fusarium oxysporum* f. sp. *niveum* | YBA | 25 |
|  | *Fusarium pseudograminearum* | YBA | 25 |
|  | *Cochliobolus miyabeanus* | PDA | 25 |
|  | *Sclerotinia sclerotiorum* | PDA | 25 |
|  | *Monilinia fructicola* | PDA | 25 |
|  | *Fusarium graminearum* | YBA | 25 |
|  | *Fusarium fujikuroi* | YBA | 25 |
|  | *Fusarium subglutinans* f. sp. *phaseoli* | YBA | 25 |
|  | *Fusarium avenaceum* | YBA | 25 |
|  | *Fusarium solani* var*.coeruleum* | YBA | 25 |
|  | *Fusarium oxysporum* f. sp. *vasinfectum* | YBA | 25 |
|  | *Magnaporthe oryzae* | PDA | 25 |
|  | *Glomerella cingulata* | PDA | 25 |
|  | *Colletotrichum gloeosporioides* | PDA | 25 |
|  | *Colletotrichum glycines* | PDA | 25 |
|  | *Colletotrichum fructicola* | PDA | 25 |
|  | *Colletotrichum lagenarium* | PDA | 25 |
|  | *Ceratocystis fimbriata* | PDA | 25 |
| Oomycetes | *Phytophthora nicotianae* | PDA | 25 |
|  | *Phytophthora capsici* | PDA | 25 |
|  | *Pythium ultimum* | PDA | 25 |
|  | *Pythium aphanidermatum* | PDA | 25 |

**Table S2**

| Isolates | Province | Location | Amount | Year | Medium | Temperature (℃) |
| --- | --- | --- | --- | --- | --- | --- |
| #41-1-5 | Liaoning | Damin tunTown, Xinmin City | 5 | 2019 | YBA | 25℃ |
| #60-1-5 | Shanxi | Shijia zhai Village, Dizhang Town, Xianyang City | 5 | 2019 |  |  |
| #73-1-5 | Shanxi | Beizhang Village, Xingjiang County, Yuncheng City | 5 | 2019 |  |  |
| #104-1-5 | Shanghai | Shanghai | 5 | 2019 |  |  |
| LY1-11 | Shandong | Caojia ying Village, Yinan su Town, Linyi City | 11 | 2014 |  |  |
| XJ1-8 |  | Xinji City, Linyi City | 8 |  |  |  |
| JZ1-15 | Shandong | Jiazhai Town, Liaocheng City | 15 | 2018 |  |  |
| TA1-15 | Shandong | Dongda wu Town, Taian City | 15 | 2018 |  |  |
| LW1-10 | Shandong | Fangxia Town, Laiwu City | 10 | 2017 |  |  |
| DZ1-12 | Shandong | Pingyuan County, Dezhou City | 12 | 2017 |  |  |
| WF1-12 | Shandong | Shouguang, Weifang City | 12 | 2017 |  |  |
| JT1-15 | Shandong | Jitai Town, Weifang City | 15 | 2018 |  |  |
| JN1-15 | Shandong | Qihe Town, Jinan City | 15 | 2018 |  |  |
| ZB1-15 | Shandong | Luwang Town, Zibo City | 15 | 2018 |  |  |
| H1-24 | Henan | Jiaozuo City | 24 | 2015 |  |  |
| H37-H52 | Shandong | Dongda wu Town, Taian City | 16 | 2017 |  |  |
| H95-H109 | Henan | Jiaozuo City | 15 | 2017 |  |  |
| H125-H135 | Shandong | Hutou Town, Linyi City | 11 | 2018 |  |  |
| H166-H175 | Shandong | Leping Town, Liaocheng City | 9 | 2018 |  |  |
| H195-H205 | Shandong | Xinji Town, Linyi City | 11 | 2018 |  |  |
| H222-H225 | Shandong | Cangshan Town, Linyi City | 4 | 2018 |  |  |
| H254-266 | Henan | Jiaozuo City | 13 | 2016 |  |  |
| 04-1,10-1 | Jiangsu | Nanjing City | 2 | 2019 |  |  |

**Table S3**

| Fungicide | Concentration (μg/mL) | |  |
| --- | --- | --- | --- |
|  | Cyclobutrifluram-sensitive isolates | Cyclobutrifluram-resistant mutants |  |
| Cyclobutrifluram | 0, 0.001, 0.005, 0.01, 0.05, 0.1 | 0, 0.01, 0.1, 0.5, 1, 5, 10 |  |
| Pydiflumetofen | 0, 0.01, 0.1, 0.5, 1, 5, 10 | 0.001, 0.005, 0.01, 0.05, 0.1 |  |
| Fluxapyroxad | 0, 0.01, 0.1, 0.5, 1, 5, 10 | 0, 0.01, 0.1, 0.5, 1, 5, 10 |  |
| Florylpicoxamid | 0, 0.01, 0.1, 1, 5, 10, 20 | 0, 0.01, 0.1, 1, 5, 10, 20 |  |
| Pyraclostrobin | 0, 0.2, 0.6, 2.5, 10, 40 | 0, 0.2, 0.6, 2.5, 10, 40 |  |
| Propineb | 0, 1, 5, 10, 15, 30 | 0, 1, 5, 10, 15, 30 |  |
| Prochloraz | 0, 0.01, 0.1, 0.5, 1, 5, 10 | 0, 0.01, 0.1, 0.5, 1, 5, 10 |  |
| Thifluzamide | 0, 0.01, 0.05, 0.1, 1, 5, 10 | 0, 0.01, 0.05, 0.1, 1, 5, 10 |  |
| Isofetamid | 0, 0.01, 0.05, 0.1, 1, 5, 10 | 0, 0.01, 0.05, 0.1, 1, 5, 10 |  |
| Isopyrazam | 0, 0.01, 0.1, 0.5, 1, 5, 10 | 0, 0.01, 0.1, 0.5, 1, 5, 10 |  |
| Penflufen | 0, 0.01, 0.1, 0.5, 1, 5, 10 | 0, 0.01, 0.1, 0.5, 1, 5, 10 |  |
| Boscalid | 0, 0.1, 0.5, 1, 5, 10, 30 | 0, 0.1, 0.5, 1, 5, 10, 30 |  |
| Fluopyram | 0, 0.01, 0.1, 0.5, 1, 5, 10 | 0, 0.01, 0.1, 0.5, 1, 5, 10 |  |

**Table S4**

| Gene | Primer | Sequence (5′-3′) | Application |
| --- | --- | --- | --- |
| *CcSdhA1* | SdhA1F | ATGCGTCGTACGCAGCTTGC | Amplification of the complete *CcSdhA1* gene |
|  | SdhA1R | GCCTTGCACTCGTTCTCATC |  |
|  | qSdhA1F | TGCGACACAACACCAAGTAC | Quantification the expression level of *CcSdhA1* gene |
|  | qSdhA1R | GCCAAAACCGTGTTCTTTGC |  |
| *CcSdhA2* | SdhA2F | ATGCGTGCCTTTGCGCGTGCCAT | Amplification of the complete *CcSdhA2* gene |
|  | SdhA2R | ACTCCTCTTCACAGGCTTCACAC |  |
|  | qSdhA2F | TTGATGTGCGGAATGGTGAG | Quantification the expression level of *CcSdhA2* gene |
|  | qSdhA2R | AATGTCCAGCAGCGAGTTTG |  |
| *CcSdhB* | SdhBF | ATGGCTTGCACACGCGCTTT | Amplification of the complete *CcSdhB* gene |
|  | SdhBR | CTACGTGAAAGCCATGCTC |  |
|  | qSdhBF | TGATGTTGGATGCGCTGATC | Quantification the expression level of *CcSdhB* gene |
|  | qSdhBR | ACGCCGTCAATGTTCATTGC |  |
| *CcSdhC* | SdhCF | ATGGCTTCCCAGCGCGTCTT | Amplification of the complete *CcSdhC* gene |
|  | SdhCR | TAAACAAACAGAGAATAGTA |  |
|  | qSdhCF | TTCTTCCACAGCTTGAACGG | Quantification the expression level of *CcSdhC* gene |
|  | qSdhCR | AAAGAGCAACGACAGACCAG |  |
| *CcSdhD* | SdhDF | ATGGCCTCCGCAATGCGCCCC | Amplification of the complete *CcSdhD* gene |
|  | SdhDR | TTAAGCGTGCCACAGCTGAG |  |
|  | qSdhDF | ATTGACTACTTCCCCGCCAAG | Quantification the expression level of *CcSdhD* gene |
|  | qSdhDR | AACGAATACAGGGCGAAACC |  |
| *CcActin* | ActF | CTCCAAGAGTGGCAAGAGCA | Quantification the expression level of *CcActin* gene |
|  | ActR | CGGTCAGATGGAAAGCTGGT |  |

**Table S5**

| Primers | Sequence (5′-3′) | Annealing temperature | Application |
| --- | --- | --- | --- |
| CcAS-278BFC | CAACTCGATGAGCATGTACCGCTGTT | 71.7℃ | Used to detect CcSdhB^H278Y^ point mutation |
| CcAS-278BR | GTGCCCACACTTAACCACGGTTCT |  |  |
| CcAS-134CFC | CATTGCGATGCCCTTCTTCTTCCTA | 68.0℃ | Used to detect CcSdhC^H134Q^ point mutation |
| CcAS-134CR | AGCCCGTCTATGAGGCGATTCTTCA |  |  |
| CcAS-135CFC | TGCCCTTCTTCTTCCACAGA | 63.6℃ | Used to detect CcSdhC^S135R^ point mutation |
| CcAS-135CR | AGCCCGTCTATGAGGCGATTCTTCA |  |  |
| CcAS-280BFC | AGCATGTACCGCTGCCACACCG | 64.0℃ | Used to detect CcSdhB^I280V^ point mutation |
| CcAS-280BR | GCAGGCCATGGCAATGGCCAT |  |  |
| CcAS-73CFA | GCAGATCACCTGGTATGCCTCAC | 65.5℃ | Used to detect CcSdhC^S73P^ point mutation |
| CcAS-73CR | ACAGTGCTTGTCACCGGTCAAG |  |  |
| CcAS-75CFT | TTGCCCTCAGCGGAGGCCTT | 60.0℃ | Used to detect CcSdhC^N75S^ point mutation |
| CcAS-75CR | GCTGTGCCAGATGTGTGCAGG |  |  |
| CcAS-134CFG | CATTGCGATGCCCTTCTTCTTCGG | 65.0℃ | Used to detect CcSdhC^H134R^ point mutation |
| CcAS-134CR | ACAGTGCTTGTCACCGGTCAAG |  |  |
| CcAS-121DFG | TCCCTCAACCCCGTCACCGGA | 65.0℃ | Used to detect CcSdhD^D121E^ point mutation |
| CcAS-121DR | AGACTGACCCCACCTCGGCGT |  |  |
| CcAS-135DFG | TCGTCATCCACTCGCACATTGT | 68.0℃ | Used to detect CcSdhD^G135V^ point mutation |
| CcAS-135DR | ACGCCGAGGTGGGGTCAGTCT |  |  |

**Table S6**

| Isolate ^*^ | Colony diameter (cm) ^#^ | | | | | | |
| --- | --- | --- | --- | --- | --- | --- | --- |
|  | 4℃ | 13℃ | 22℃ | 25℃ | 28℃ | 30℃ | 37℃ |
| **H1** | 1.87 ± 0.044 c | 2.36 ± 0.102 c | 5.38 ± 0.014 b | 6.96 ± 0.019 b | 6.94 ± 0.011 b | 7.03 ± 0.027 b | 3.32 ± 0.062 a |
| H1-1-5 | 2.25 ± 0.046 b | 2.54 ± 0.051 b | 4.72 ± 0.096 c | 6.06 ± 0.069 c | 5.87 ± 0.051 c | 5.88 ± 0.020 c | 1.05 ± 0.021 c |
| H1-2-4 | 2.80 ± 0.061 a | 2.73 ± 0.012 a | 5.92 ± 0.045 a | 7.40 ± 0.024 a | 7.56 ± 0.032 a | 7.53 ± 0.007 a | 1.93 ± 0.011 b |
| **H105** | 2.01 ± 0.060 b | 2.68 ± 0.036 b | 5.95 ± 0.009 a | 7.56 ± 0.034 a | 7.76 ± 0.014 a | 7.68 ± 0.009 a | 3.67 ± 0.015 a |
| H105-1-3 | 2.56 ± 0.056 a | 2.94 ± 0.034 a | 5.81 ± 0.027 a | 7.55 ± 0.017 a | 7.71 ± 0.005 a | 7.63 ± 0.006 a | 3.52 ± 0.016 b |
| H105-3-2 | 1.63 ± 0.008 c | 2.29 ± 0.051 c | 3.98 ± 0.102 b | 5.35 ± 0.018 b | 5.51 ± 0.036 b | 5.37 ± 0.067 b | 1.64 ± 0.038 c |
| **H262** | 1.19 ± 0.030 b | 2.47 ± 0.029 b | 4.94 ± 0.024 a | 7.17 ± 0.171 b | 7.78 ± 0.071 a | 7.99 ± 0.022 a | 3.98 ± 0.090 a |
| H262-2-8 | 1.85 ± 0.027 a | 2.87 ± 0.012 a | 5.19 ± 0.112 a | 7.72 ± 0.034 a | 7.83 ± 0.033 a | 7.57 ± 0.123 b | 4.12 ± 0.014 a |

**Table S7**

| Isolate | Mutation type | Nucleotide change | Codon change | Amino acid change |
| --- | --- | --- | --- | --- |
| #104-3 | CcSdhB^H278Y^ | C832T | CAC→TAC | His (H)→Tyr (Y) |
| H107 | CcSdhB^I280V^ | A838G | ATT→GTT | Ile (I)→Val (V) |
| #41-2 | CcSdhC^S73P^ | T217C | TCG→CCG | Ser (S)→Pro (P) |
| #41-3 | CcSdhC^N75S^ | A224G | AAC→AGC | Asn (N)→Ser (S) |
| H255 | CcSdhC^H134R^ | A401G | CAC→CGC | His (H)→Arg (R) |
| H1-1-5 | CcSdhC^H134Q^ | C402A | CAC→CAA | His (H)→Gln (Q) |
| H105-3-2 | CcSdhC^S135R^ | C405A | AGC→AGA | Ser (S)→Arg (R) |
| H97 | CcSdhD^D121E^ | C363A | GAC→GAA | Asp (D)→Glu (E) |
| #41-5 | CcSdhD^G135V^ | G404T | GGC→GTC | Gly (G)→Val (V) |

**Table S8**

| Mutation type | Number of isolates^*^ | Frequency (%) |
| --- | --- | --- |
| CcSdhB^H278Y^ | 20 | 7.9 |
| CcSdhB^I280V^ | 52 | 20.6 |
| CcSdhC^S73P^ | 51 | 20.2 |
| CcSdhC^N75S^ | 11 | 4.3 |
| CcSdhC^H134R^ | 23 | 9.1 |
| CcSdhD^D121E^ | 11 | 4.3 |
| CcSdhD^G135V^ | 19 | 7.5 |

**Fig. S1**


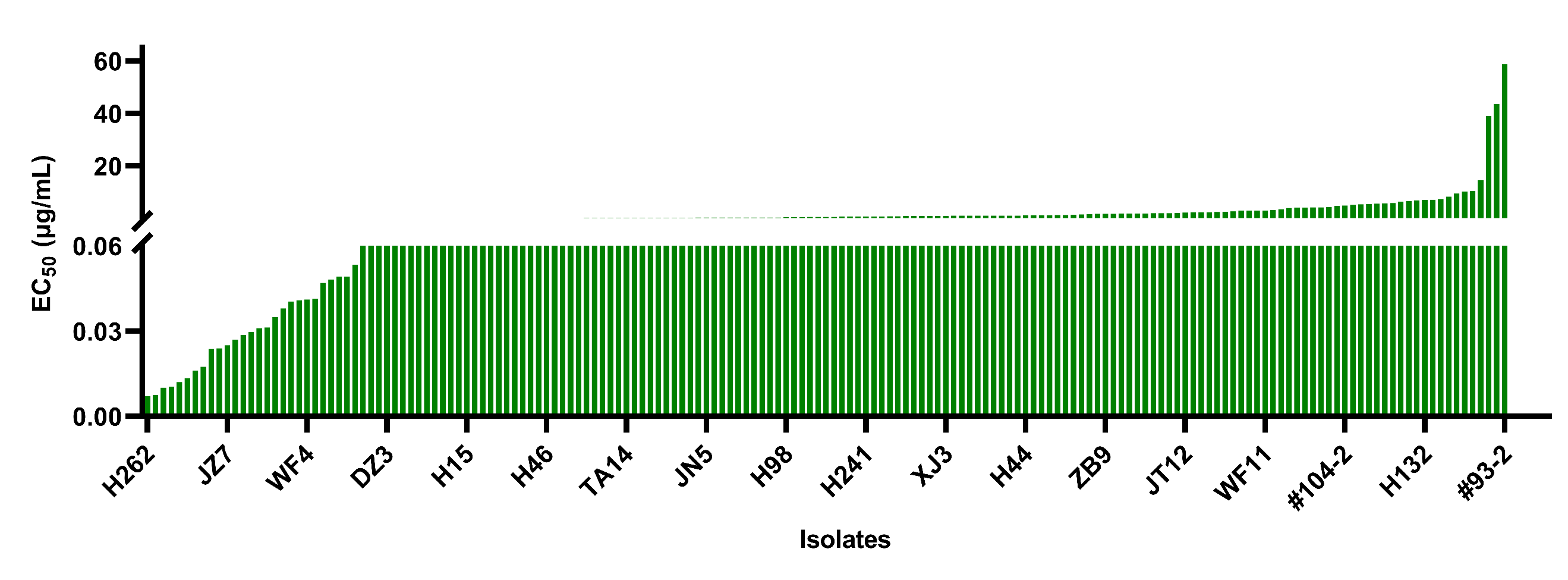


**Fig. S2**


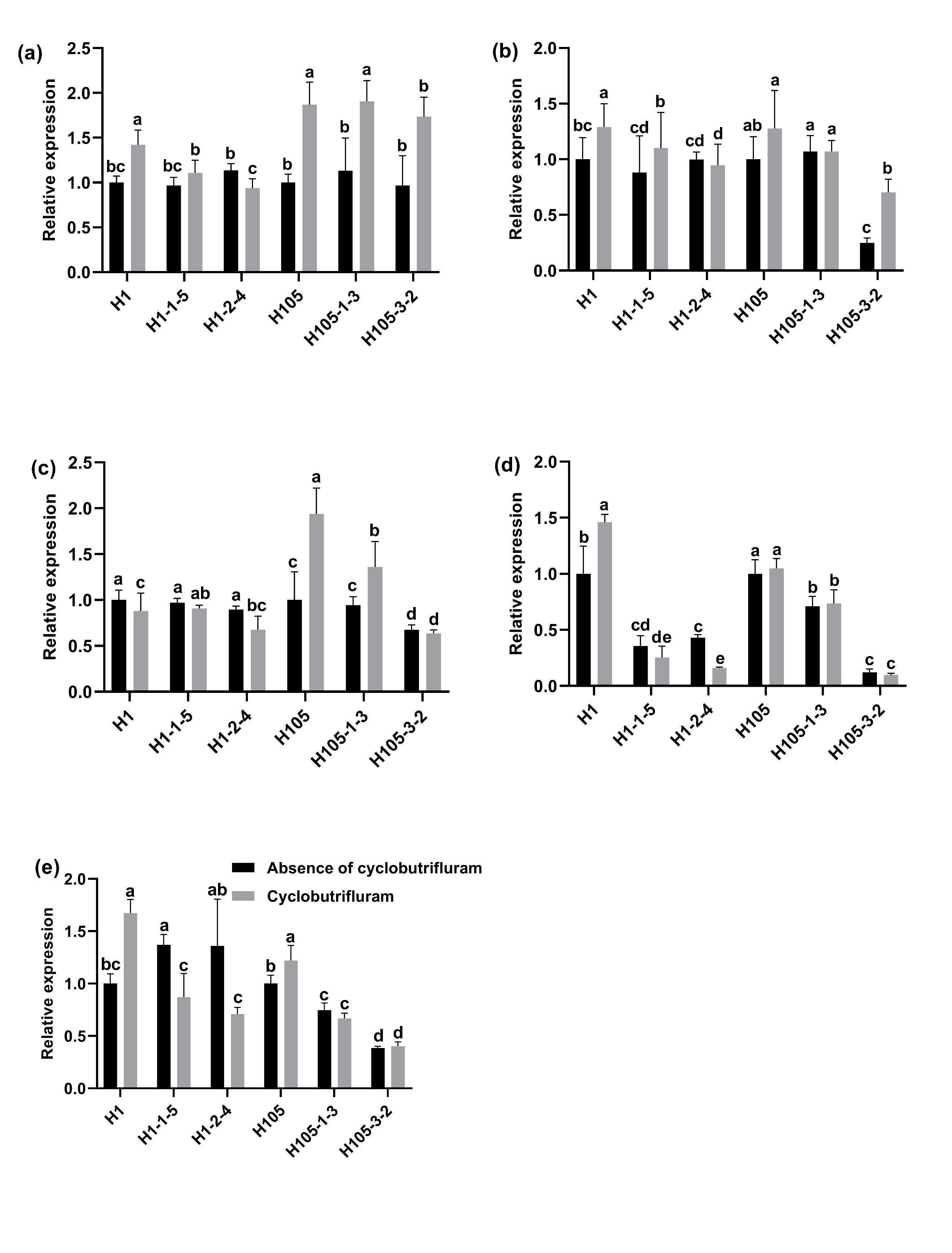

Supplement: Supplementary file 1 — Supplementary Material 1: Table S1 Pathogens used in this study. Table S2 Information on Corynespora cassiicola isolates collected from 2014 to 2019. Table S3 Concentrations used to determine the sensitivity to different fungicides of wild-type isolates and cyclobutrifluram-resistant mutants of Corynespora cassiicola. Table S4 Primers used to amplify and quantify the CcSdh genes in this study. Table S5 AS-PCR primers used to detect cyclobutrifluram-resistant isolates of Corynespora cassiicola. Table S6 Mycelial growth of Corynespora cassiicola isolates at various temperatures on PDA plates. *Isolates in bold are parents of the resistant mutants; #Mean ± standard error in a column followed by the same letter means there is no significant difference in ANOVA with Tukey’s HSD test at P < 0.05. Table S7 Information on point mutations in this study. Table S8 Detection by AS-PCR of cyclobutrifluram-resistant Corynespora cassiicola isolates collected from the field. *253 C. cassiicola isolates collected from the field were used in the AS-PCR detection. Fig. S1 EC50 values of 171 Corynespora cassiicola isolates to cyclobutrifluram. Fig. S2 Expression levels of CcSdh genes in cyclobutrifluram-resistant mutants of Corynespora cassiicola and their parental isolates. Transcript levels were normalized to the expression levels of CcSdh genes in the sensitive isolate H1 in the absence of fungicide: (a) CcSdhA1, (b) CcSdhA2, (c) CcSdhB, (d) CcSdhC, and (e) CcSdhD. [file 44154_2025_251_MOESM1_ESM.docx]
